# Jerky Movement with Ceftazidime: A Case of Ceftazidime-Induced Neurotoxicity with a Review of the Literature

**DOI:** 10.1155/2019/8936478

**Published:** 2019-12-12

**Authors:** Mohammed Al-Sadawi, Romy Rodriguez Ortega, Natalie Sun, Madina Abdurahimova, Samy I. McFarlane

**Affiliations:** Department of Internal Medicine, State University of New York: Downstate Medical Center, Brooklyn, NY 11203, USA

## Abstract

Neurotoxicity manifested as confusion and seizures has been recognized as a side effect of multiple cephalosporins including ceftazidime. Renal impairment and inappropriate dosing are the most common contributors to the development of neurological abnormalities in patients receiving these antibiotics. The presence of baseline neurological abnormalities likely contributes to the frequency of these adverse events. Here, we present a case of a 78-year-old man that developed altered mental status and myoclonic movement after initiation of ceftazidime in the setting of mild renal dysfunction. Resolution of clinical picture was evident after 48 hours of discontinuation of the antibiotic without additional interventions.

## 1. Introduction

Cephalosporins are among the most commonly prescribed classes of antibiotics. They provide wide antimicrobial coverage and a relatively low incidence of side effects. They are also widely available, and most clinicians have extensive experience in their use. The most common side effect of cephalosporins as a group is hypersensitivity reactions more commonly characterized by skin reactions with occasional systemic symptoms. Ceftazidime is a third-generation cephalosporin with antipseudomonal activity, and as consequence commonly used when Gram-negative coverage including pseudomonal antimicrobial activity is needed. Ceftazidime has excellent CSF penetration and can be used to treat CNS infections, but this pharmacokinetic property also opens the door for rare cases of neurotoxicity [1]. Other cephalosporins able to cross the blood-brain barrier also exhibit this side effect profile that appears to be caused by inhibition of the gamma-aminobutyric acid (GABA) receptor [2]. The most commonly reported cephalosporin as a cause of neurotoxicity is cefepime, but ceftazidime has also been implicated in multiple case reports.

Cephalosporin-induced neurotoxicity is usually characterized by confusion, seizures, or myoclonus in patients with renal impairment. This is particularly true in the setting of renal impairment though cases also exist in those with normal creatinine clearance. Previous CNS disease has also been suggested as decreasing the threshold of nervous system toxicity with the use of third- and fourth-generation cephalosporin [3]. In addition to preexisting CNS conditions, reduced creatinine clearance, impaired renal function, and excess dosage of medication have been described as independent risk factors for neurotoxic effects [4]. The typical time period for encephalopathy induced by cephalosporin use is a latency of 2 to 10 days following the start of medication and resolution in 2 to 7 days following discontinuation [5]. Usually, withdrawal of the antibiotic and supportive care is sufficient for clinical improvement [6].

Here, we present the case of a 78-year-old man that while being treated for a complicated urinary tract infection with the antibiotic ceftazidime developed myoclonic movements and neurological deficits in the setting of worsening renal function.

## 2. Case Presentation

**Figure 1:**
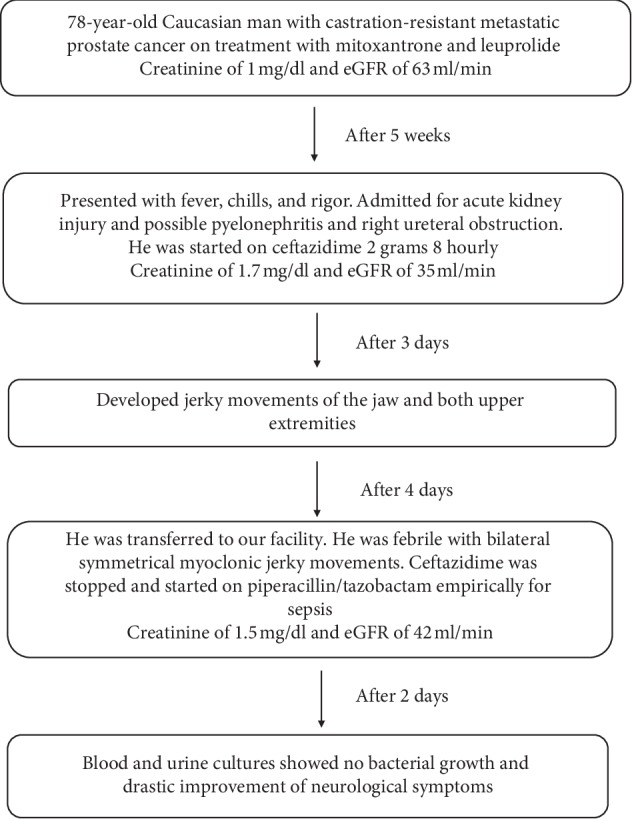
Timeline of case presentation.

A 78-year-old Caucasian man with a past medical history of peripheral neuropathy, stool incontinence, benign prostatic hyperplasia, chronic urinary retention with frequent self-catheterization, and Gleason 4 + 5 (9) castration-resistant metastatic prostate cancer to the lymph nodes, lungs, bone, brain, and liver, currently on treatment with mitoxantrone and leuprolide was transferred from a different hospital for concerns over fever and new abnormal movements. One week before transfer, the patient experienced fever, chills, and rigors. Laboratory tests and blood, urine, and stool cultures were sent along with chest X-ray CT scan of the head, abdomen, and pelvis. He was treated at the transferring institution with ceftazidime 2 grams every 8 hours for a provisional diagnosis of pyelonephritis and right ureteral obstruction. Laboratory tests showed elevated creatinine to 1.71 mg/dl (normal 0.7–1) and negative cultures. CT scan showed unchanged metastases in the brain, abdomen, and pelvis. On day 3 of admission, he developed jerky movements of the jaw and both upper extremities. When he reached our facility, he was sleepy, oriented only to person. He was febrile to 101.9 F with a blood pressure of 100/65, a heart rate of 87, and oxygen saturation at room air 96% with good airway protection. He had bilateral symmetrical myoclonic jerky movements of the jaw and arms interfering with swallowing, talking, and fine hand activities. Neurological examination was otherwise unremarkable. His initial laboratory results at our facility showed creatinine of 1.5 mg/dl (normal 0.7–1) and estimated glomerular filtration rate of 42 ml/min. His baseline kidney function 5 weeks before current presentation was a creatinine level of 1 mg/dl and estimated glomerular filtration rate of 63 ml/min. Ceftazidime was stopped, and he was started on piperacillin/tazobactam empirically for sepsis, it was the provisional diagnosis given the disturbed conscious level, fever, and the patient is on chemotherapy. He was given dexamethasone for suspicion of worsening brain metastases. Magnetic resonance of the brain and spine showed stable unchanged metastases in the cerebellum and left choroids and no signs of infection or inflammatory process, and based on these findings, steroids were discontinued. Thyroid function tests, ammonia, and vitamin B12 and folate levels were within the normal range. Electroencephalogram demonstrated global symmetrical hypoactivity. After 48 hours, blood and urine cultures showed no bacterial growth and drastic improvement of neurological symptoms. Computed tomography of the abdomen and pelvis showed no signs of infection or acute pathology. He was able to swallow and talk without limitation and self-catheterize. Antibiotics were discontinued, and he demonstrated clinical improvement without further specific therapy. Upon clinical improvement, the patient was discharged to a rehabilitation facility due to deconditioning from his hospital course with no changes in neurological function from baseline (Figure 1).

## 3. Discussion

Cephalosporin-induced neurotoxicity has been increasingly reported in the literature mainly in the setting of renal impairment. The most commonly reported cephalosporin as a cause of neurological adverse effects has been cefepime, but ceftazidime has also been implicated [6]. Multiple systematic analyses have reported the incidence and clinical profile of these events. Neurological adverse events significant to warrant discontinuation of therapy were found in 0.2% of patients treated with cefepime and 0.3% of patients treated with ceftazidime in a study evaluating the safety of cefepime [7]. A recent publication of an adverse drug reactions monitoring program in France reported that the most commonly implicated cephalosporins in neurotoxicity were cefepime followed by ceftriaxone and ceftazidime [8].

In addition of elevated levels of cephalosporins in case of renal dysfunction, elevated blood urea nitrogen glycated and carbamylated proteins increased the permeability of the blood-brain barrier [9–11]. The pathophysiology of neurotoxicity includes decreased inhibitory gamma-aminobutyric acid (GABA) and increased excitatory amino acid release [2, 12]. On the contrary, cephalosporins increased endotoxins and cytokines which stimulated amino acid release [2].

The mechanism responsible for cephalosporin-mediated neurotoxicity is predominantly associated with the inhibition of the GABAA receptor function. This specific mechanism appears to be specially associated with the capacity of cephalosporins to induce convulsions. Cefepime has been demonstrated to have greater affinity for the GABAA receptor than ceftazidime [13]. The risk for neurotoxicity is significantly increased in the setting of renal failure and decreased the clearance of cephalosporins. This has been demonstrated in animal studies with renal failure models and is evident in reviews from human cases [6, 14].

The most common reported clinical manifestations are confusion, seizures, and myoclonus. Ceftazidime in particular has been more commonly associated with confusion and myoclonus than with seizures [13]. The manifestations in this specific case were in keeping with this report, as our patient upon transfer presented with confusion and myoclonic movements. The timing of neurological manifestations after initiation of ceftazidime although consistent with cephalosporin-induced neurotoxicity could have been the result of other pathological process. The median duration of therapy before the onset of symptoms has been reported to be around 5 days [13]. Here, the first dose of ceftazidime occurred 3 days before symptom onset. Metastatic brain lesion progression from prostate cancer was considered, but magnetic resonance imaging did not reveal any changes to these lesions, or any new findings (stroke and demyelination) that could justify this particular syndrome. The presence of baseline abnormalities could have contributed to increased susceptibility to CNS side effects as a result of exposure to cephalosporins. One case series has reported neurotoxicity in the setting of previous neurological disease and renal impairment [15]. Worsening of the underlying urinary tract infection causing toxic-metabolic encephalopathy due to sepsis was another consideration, but workup including hemogram, urinalysis, chest, abdominal and pelvic imaging, and culture results was not indicative of any infectious source at the time. However, the patient was continued on antibiotics initially in our facility due to fever, relative immunocompromised state, borderline blood pressure, and neurological symptoms.

Probably, the strongest factor that contributed to the suspicion for ceftazidime-induced neurotoxicity was the drastic improvement of his clinical condition within 48 hours of discontinuation of the antibiotic. Duration of symptoms secondary to ceftazidime toxicity have been found to be shorter when compared to cefepime (average 4 days versus 8 days) [13], but resolution of symptoms as early as 36 hours in an elderly patient with normal renal function has been reported in the literature [16]. Our patient had evidence of acute kidney injury that was mild or stage 1 (AKIN/KDIGO) given that the increase in creatinine from baseline was <100% and there was no evidence of decreased urinary output. Based on this, the time to resolution of symptoms would be consistent with cephalosporin-induced neurotoxicity. Another factor that likely contributed to this adverse drug effect is that ceftazidime was not adjusted based on creatinine clearance which for this patient would have been 1 gram every 12 hours, instead of 2 grams.

Granted that the proposed mechanism for neurotoxicity is GABA mediated, benzodiazepines have been proposed as first-line therapy, mainly in the setting of status epilepticus [17]. But most scenarios of neurological symptoms have resolved with only discontinuation of the offending agent. Prevention, by appropriate dosing and consideration of renal function, is likely to have the biggest impact. In cases of severe neurological compromise as characterized by refractory seizures and coma, removal of cephalosporins by hemodialysis is supported by at least one case series, in which 4 out of 5 patients improved after hemodialysis [18]. Our patient improved after discontinuation of antibiotic therapy, and no subsequent treatment was required. Populations at risk of neurotoxicity associated with various groups of antibiotics include those with extremes of age, critical illness, renal dysfunction, and prior neurological disease [19].

## 4. Conclusion

Cephalosporin and specifically ceftazidime-induced neurotoxicity although an infrequent adverse effect is likely underrecognized especially in extremes of age, critical illness, renal dysfunction, and prior neurological disease. Appropriate dosing and recognition of the impact of renal dysfunction are likely to significantly reduce the incidence of this event. Although in some cases more aggressive treatment might be required (antiepileptic treatment or dialysis), in general early recognition and discontinuation of the offending agent result in clinical improvement.

## Additional Points

*Learning Points*. (i) Cephalosporins can be associated with neurological manifestation especially in the old population with kidney dysfunction. (ii) Cephalosporins should be renally dosed to avoid cephalosporin-related neurological manifestation.
